# Detection of Apple Leaf Diseases Based on LightYOLO-AppleLeafDx

**DOI:** 10.3390/plants14040599

**Published:** 2025-02-17

**Authors:** Hongyan Zou, Peng Lv, Maocheng Zhao

**Affiliations:** College of Mechanical and Electronic Engineering, Nanjing Forestry University, Nanjing 210037, China; zouhy@njfu.edu.cn (H.Z.); penglv@njfu.edu.cn (P.L.)

**Keywords:** leaf diseases, convolutional neural networks, deep learning, YOLOv8, slim neck, lightweight, SPD-Conv

## Abstract

Early detection of apple leaf diseases is essential for enhancing orchard management efficiency and crop yield. This study introduces LightYOLO-AppleLeafDx, a lightweight detection framework based on an improved YOLOv8 model. Key enhancements include the incorporation of Slim-Neck, SPD-Conv, and SAHead modules, which optimize the model’s structure to improve detection accuracy and recall while significantly reducing the number of parameters and computational complexity. Ablation studies validate the positive impact of these modules on model performance. The final LightYOLO-AppleLeafDx achieves a precision of 0.930, mAP@0.5 of 0.965, and mAP@0.5:0.95 of 0.587, surpassing the original YOLOv8n and other benchmark models. The model is highly lightweight, with a size of only 5.2 MB, and supports real-time detection at 107.2 frames per second. When deployed on an RV1103 hardware platform via an NPU-compatible framework, it maintains a detection speed of 14.8 frames per second, demonstrating practical applicability. These results highlight the potential of LightYOLO-AppleLeafDx as an efficient and lightweight solution for precision agriculture, addressing the need for accurate and real-time apple leaf disease detection.

## 1. Introduction

Apples are the most produced fruit in China, with the planting area consistently exceeding 29 million mu (approximately 1.93 million hectares) over the past decade and an annual production nearing 50 million tons. In 2022, China’s apple production reached 47.57 million tons, accounting for 15.2% of the nation’s total fruit output of 312.96 million tons [1,2]. However, apple leaf diseases significantly impact the productivity and sustainability of the apple industry in China. Common diseases such as speckle leaf drop, brown spot, grey spot, mosaic, and rust cause symptoms like yellowing leaves and, in severe cases, lead to leaf defoliation. These conditions hinder tree vigor, reduce resistance to other diseases, and ultimately lower fruit yield. The economic losses escalate as diseases spread [3,4].

Research in plant leaf disease detection has evolved from traditional image processing and machine learning to convolutional neural network (CNN)-based deep learning models. In Omrani et al. [5], three common diseases of apple leaves were selected as analysis targets, features were extracted, and diseases were classified using a support vector machine (SVM). In 2015, the introduction of Faster R-CNN provided new solutions for real-time object detection [6]. Subsequently, methods such as YOLOv5, U-Net, and ResNet have been extensively applied to identify and segment apple leaf diseases, achieving high detection accuracy and real-time performance [7,8]. For instance, Jiang et al. [9] proposed the INAR-SSD model based on GoogLeNet Inception and Rainbow concatenation, achieving a mean average precision (mAP) of 78.80% with a detection speed of 23.13 FPS on the ALDD dataset. Chao et al. [10] combined DenseNet and Xception with global average pooling, achieving an overall accuracy of 98.82%. However, many models remain hindered by large sizes and complex architectures, making deployment challenging. Large-scale models demand substantial computational resources and memory, which conflicts with the limited hardware capabilities of edge devices commonly used in agricultural settings. High computational complexity further leads to increased power consumption and latency, hindering real-time disease monitoring in orchards. Additionally, oversized models face compatibility issues when integrated with existing agricultural IoT platforms, limiting their practical adoption by farmers and agricultural technicians.

Lightweight techniques for apple leaf disease detection continue to be an area of active research. Liu et al. [11] introduced asymmetric ShuffleBlocks, CSP modules, and BSConv, achieving an mAP of 91.08% on the MSALDD dataset and 58.85% on the PlantDoc dataset at 122 FPS. Xu et al. [12] compressed the model size by replacing common convolution with group convolution in the space pyramid pool cross-stage partial convolution (SPPCSPC) module, achieved 90.2% accuracy on the test set, and reduced floating-point operations (FLOPs) to 6.1 GFLOPs/G. Wang et al. [13] reduced parameters by using Ghost modules and improved feature extraction with Mobile Inverted Residual Bottleneck Convolution and CBAM, achieving a maximum mAP of 89.3% with an FPS of 84.1. Zhu et al. [14] enhanced accuracy by incorporating feature enhancement modules and coordinate attention, with Apple-Net achieving an mAP@0.5 of 95.9% and a precision of 93.1%.

To address the limitations of large model size and computational complexity, this study proposes LightYOLO-AppleLeafDx, a lightweight model based on YOLOv8, for efficient apple leaf disease detection. The main innovations include (1) SPD-Conv, a novel spatial-depth conversion convolution technique, which improves small-object detection and low-resolution image processing by reducing information loss and enhancing feature extraction accuracy; (2) a Slim-Neck architecture to build an efficient neural network backbone; and (3) a self-attention-based lightweight detection head to reduce computational complexity. These improvements enable deployment on edge devices such as the RV1103 while maintaining robust detection performance.

## 2. Materials and Methods

### 2.1. Materials

**Figure 1:**
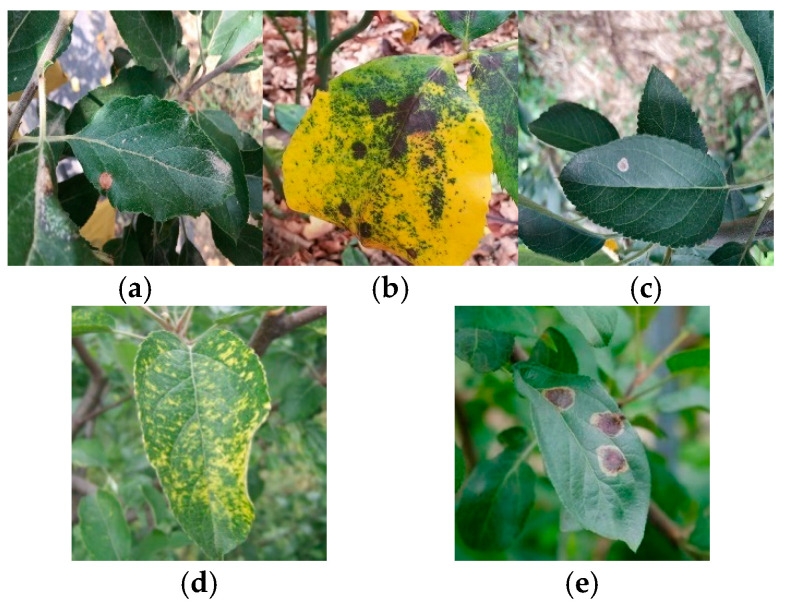
Example of apple leaf diseases. (**a**) Spot disease, (**b**) brown spot, (**c**) grey spot, (**d**) mosaic disease, (**e**) rust disease.

The experimental dataset was derived from the SCIDB public dataset (Apple Tree Leaf Disease Segmentation Dataset) and the Kaggle public dataset (Apple Leaf Disease Dataset). After manual curation, images of five common types of apple leaf diseases were selected to form the initial dataset. Examples of these diseases are shown in Figure 1, illustrating the typical appearance and variations within the dataset. All disease instances were annotated using axis-aligned bounding boxes in YOLO format (class_id x_center y_center width height), where coordinates and dimensions are normalized to [0, 1]. To enhance the dataset’s utility, the open-source annotation tool Labelme was used to manually annotate disease images, generating corresponding TXT label files [15].

Data augmentation [16,17], a widely used and effective technique in computer vision, is particularly beneficial for tasks like object detection in YOLO. By generating diverse training samples, data augmentation enhances model generalization capabilities [18]. The imgaug library was employed to expand the dataset using transformations such as rotation, translation, scaling, flipping, cropping, color jittering, and noise addition [19]. Finally, the dataset was split into training and testing subsets using the sklearn library, with 80% of the data allocated for training and 20% for testing. To ensure reproducibility, fixed random seeds (seed = 1) were applied during the data splitting process. The details of the dataset are shown in Table 1.

### 2.2. Methods

YOLO (You Only Look Once) is a real-time object detection algorithm based on a single-stage detection approach, which integrates object classification and localization regression within a single forward pass of a neural network [20,21,22]. YOLOv8 (You Only Look Once, version 8) is the latest iteration in the YOLO series of single-stage convolutional neural network models, widely applied in object detection tasks [23]. Compared to its predecessors, YOLOv8 offers improved precision and inference speed while optimizing its architecture for efficient deployment on mobile and embedded devices [24].

YOLOv8 performs end-to-end object detection by employing a single neural network to directly generate object bounding boxes and class probabilities from input images. Its architecture consists of four main components: the input layer, backbone network, feature pyramid network (FPN + PAN), and detection head [25].

The backbone network is responsible for extracting essential features from the input images and incorporates an enhanced CSPDarknet53 (Cross Stage Partial Network 53) structure. This improvement reduces computational cost while retaining more feature information, improves gradient flow, and enhances the model’s convergence speed and detection performance [26].

The model leverages enhanced versions of the FPN (feature pyramid network) and PAN (path aggregation network) to better capture multi-scale targets. FPN facilitates the fusion of feature maps from different layers, improving the detection of small objects, while PAN optimizes the propagation of feature information, strengthening the influence of high-level features on detection results.

Finally, the detection head is responsible for generating bounding boxes and classification results. Similar to its predecessors, it utilizes anchor boxes for predicting object locations and adopts a multi-scale prediction mechanism to enhance detection performance across objects of varying sizes [27,28].

These advancements position YOLOv8 as a powerful and versatile solution for object detection tasks across diverse application scenarios.

#### 2.2.1. Proposed Algorithm

**Figure 2:**
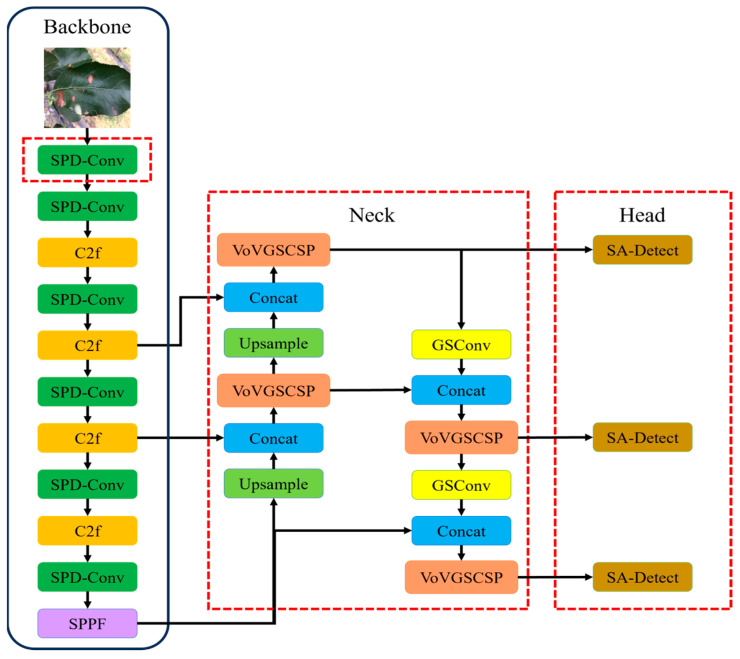
LightYOLO-AppleLeafDx model.

Based on the characteristics of the YOLOv8 model, an improved detection model for apple leaf diseases, named LightYOLO-AppleLeafDx, was developed to enhance detection accuracy, particularly for small-scale leaf diseases in complex backgrounds, as illustrated in Figure 2.

To achieve this, the backbone network was improved by replacing the standard convolution (Conv) layers with SPD-Conv modules, which utilize spatial-depth transformation to enhance feature extraction efficiency and improve the model’s capability in handling small objects and low-resolution images. For the Neck network, the standard convolution and C2f modules were substituted with a lightweight GSConv module and an aggregated VoV-GSCSP structure, forming a Slim-Neck architecture. This design balances feature extraction performance while significantly reducing model complexity and computational costs.

In addition, the original detection head was refined by introducing a Self-Attention Module and a Convolution Module, designed to optimize feature weighting and reduce computational overhead. These modifications collectively streamline the architecture and enhance the model’s generalizability.

The resulting LightYOLO-AppleLeafDx model integrates these improvements, offering a lightweight yet effective solution for detecting apple leaf diseases under real-world conditions while ensuring high inference speed and accuracy.

#### 2.2.2. Improvements in the Convolutional Neural Network

Convolutional neural networks (CNNs) often suffer from fine-grained information loss and inefficient feature learning due to the use of traditional strided convolution and pooling layers [29]. To address these issues, the SPD-Conv (Space-to-Depth Convolution) technique has been introduced as a replacement for strided convolution and pooling layers. SPD-Conv transforms spatial information into depth information, allowing CNNs to learn image features more effectively [30]. This approach reduces information loss, enhances feature extraction accuracy, and optimizes the model’s ability to process small objects and low-resolution images.

Assume an intermediate feature map X of size $S\times S\times C_{1}$. A series of sub-feature maps is generated through slicing as follows:

$$\begin{array}{ll} & \begin{array}{l} f_{0,0}=X\left[0:S:scale,0:S:scale\right],\;f_{1,0}=X\left[1:S:scale,0:S:scale\right],\ldots , \end{array}\\ & \begin{array}{l} f_{scale\;-\;1,0}=X\left[scale\;-\;1:S:scale,0:S:scale\right]; \end{array}\\ & f_{0,1}=X\left[0:S:scale,1:S:scale\right],\;f_{1,1},\ldots ,\\ & f_{scale\;-\;1,1}=X\left[scale\;-\;1:S:scale,0:S:scale\right];\\ & \vdots \\ & f_{0,scale-1}=X\left[0:S:scale,scale-1:S:scale\right],\;f_{1,scale\;-1},\ldots ,\\ & f_{scale-1,scale-1}=X\left[scale-1:S:scale,scale-1:S:scale\right]. \end{array}$$

**Figure 3:**
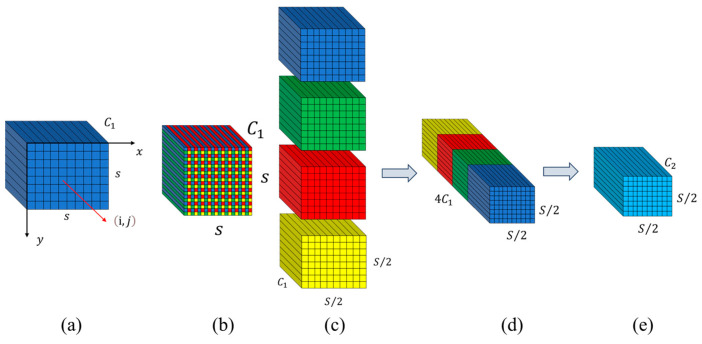
SPD-Conv structure diagram when scale=2. (**a**) Input Feature Map, (**b**) Sub-feature Map Extraction, (**c**) Sub-feature Map Concatenation, (**d**) Non-strided Convolution, (**e**) Output Feature Map.

Given any original feature map X, a sub-feature map $f_{x,y}$ is generated by selecting all elements $X\left(i,j\right)$ where i+x and j+y are divisible by scale. Consequently, each sub-feature map is downsampled by a factor of scale relative to X. When scale=2, as shown in Figure 3a–c, four sub-feature maps $f_{0,0}$, $f_{1,0}$, $f_{0,1}$, and $f_{1,1}$ are obtained. Each sub-feature map has a size of $\left(\frac{S}{2},\frac{S}{2},C_{1}\right)$ and is downsampled by a factor of 2 relative to the original feature map X.

Subsequently, these sub-feature maps are concatenated along the channel dimension to produce a feature map $X^{\prime }$. At this stage, the spatial dimensions of the feature map are reduced by a factor of scale, while the channel dimension is increased by a factor of $scale^{2}$. As shown in Figure 3d, SPD transforms the feature map $X\left(S,S,C_{1}\right)$ into an intermediate feature map $X^{\prime }\left(\frac{S}{scale},\frac{S}{scale},scale^{2}C_{1}\right)$. After the SPD transformation, as shown in Figure 3e, we have a non-strided (i.e., stride = 1) convolution layer with $C_{2}$ filters where $C_{2}<scale^{2}C_{1}$ and the operation is expressed as $X^{\prime }\left(\frac{S}{scale},\frac{S}{scale},scale^{2}\cdot C_{1}\right)\to X^{\prime \prime }\left(\frac{S}{scale},\frac{S}{scale},C_{2}\right)$.

In this study, the standard convolution layers in the Backbone of YOLOv8 were replaced with the SPD-Conv module. This substitution significantly reduces computational complexity without compromising model accuracy, enhancing the network’s efficiency in real-world applications.

#### 2.2.3. Improvements in the Neck Component

The Slim-Neck is an optimized design for the neck component of convolutional neural networks (CNNs), which serves as the bridge between the Backbone and Head networks in object detection frameworks. The neck is responsible for feature fusion and processing, playing a crucial role in improving detection accuracy and efficiency [31]. The design of the Slim-Neck is inspired by advancements in DenseNet [32] and CSPNet [33].

**Figure 4:**
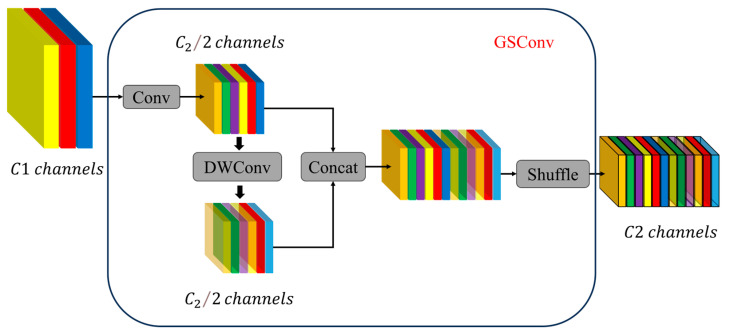
GSConv network structure.

To enhance efficiency, the Slim-Neck replaces standard convolutions with lightweight GSConv. GSConv combines standard convolution (Conv) and depthwise separable convolution (DWConv), which helps mitigate the semantic information loss caused by progressive spatial-to-channel transformation. This hybrid convolution maximizes the retention of implicit links between channels, as illustrated in Figure 4.

**Figure 5:**
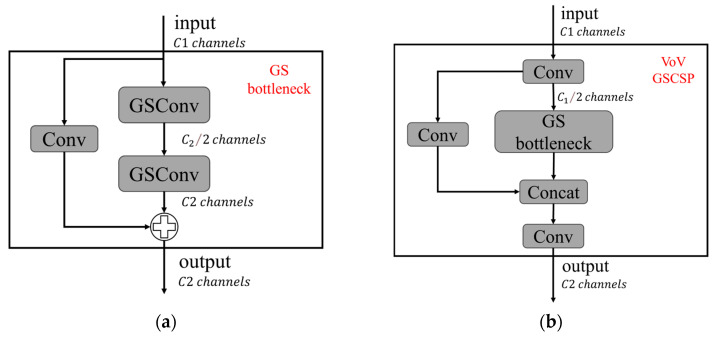
Structure of the VoV-GSCSP module. (**a**) GS Bottleneck, (**b**) VoV-GSCSP.

Building on GSConv, the GS Bottleneck (Figure 5a) is introduced as a foundational building block for the Slim-Neck architecture. Additionally, the VoV-GSCSP [31] module (Figure 5b) leverages a one-time aggregation strategy for cross-stage partial network integration. This design ensures efficient information fusion between feature maps at different stages, improving the model’s ability to handle multi-scale features while maintaining computational efficiency.

These enhancements to the neck architecture contribute to improved feature processing, enabling the proposed model to achieve high detection performance with reduced complexity and resource demands.

#### 2.2.4. Improvements in the Detection Head

**Figure 6:**
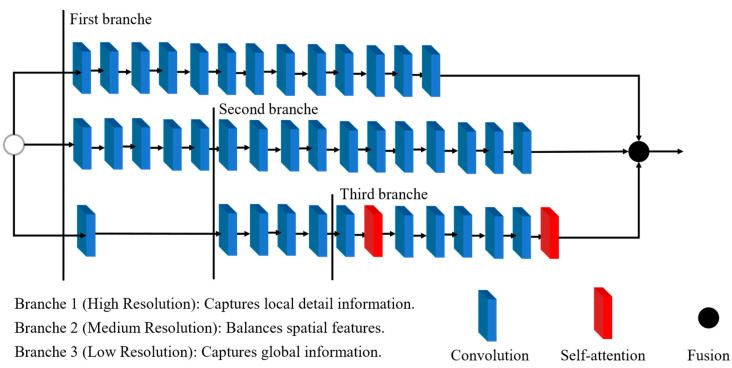
Lightweight Self-Attention detection head.

The detection head plays a critical role in generating object bounding boxes and classification results. To enhance its efficiency, this study draws inspiration from the Hybrid Convolutional-Transformer Architecture Search (HyCTAS) method, which leverages genetic algorithms to optimize and automatically construct network architectures. HyCTAS effectively integrates self-attention mechanisms into convolutional neural networks, as illustrated in Figure 6 [34].

The detection head incorporates two key components: the Self-Attention Module and the Convolution Module, designed to strike an optimal balance between feature extraction efficiency and computational cost.

**Figure 7:**
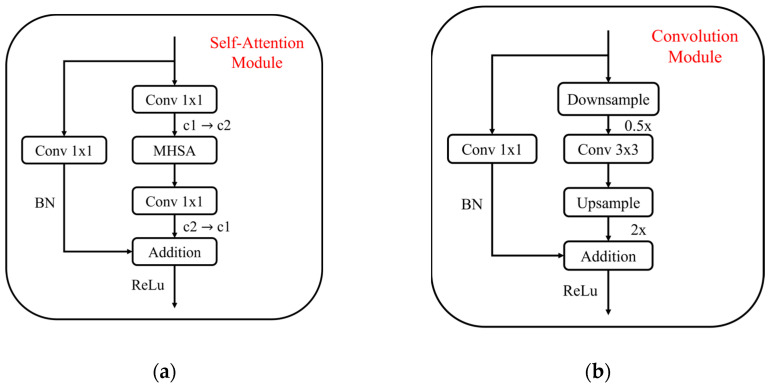
Combination module. (**a**) Self-Attention Module, (**b**) Convolution Module.

Self-Attention Module (Figure 7a): This module employs multi-head self-attention (MHSA) layers to capture broad contextual information. It combines MHSA with 1 × 1 convolution layers, batch normalization (BN), residual connections, and ReLU activation functions, ensuring effective feature learning while maintaining robust performance.

Convolution Module (Figure 7b): This module focuses on preserving local information through downsampling and upsampling operations. It reduces computational complexity by performing frequency reduction and feature transformation.

The synergy between these modules enables the detection head to effectively extract global and local features while maintaining computational efficiency. This combination facilitates the architecture search process to identify a structure that achieves high detection accuracy with minimal resource demands.

## 3. Results

### 3.1. Experimental Environment and Model Evaluation Metrics

The experiments in this study were conducted on a system running Windows 10 Professional (64-bit) with an Intel(R) Core(TM) i3-12100F CPU @ 3.30 GHz and a NVIDIA GeForce RTX 2080 Ti GPU. The software environment comprised Python 3.8, PyTorch 2.3.1, and CUDA 11.8. Detailed experimental hyperparameters are listed in Table 2.

**Figure 8:**
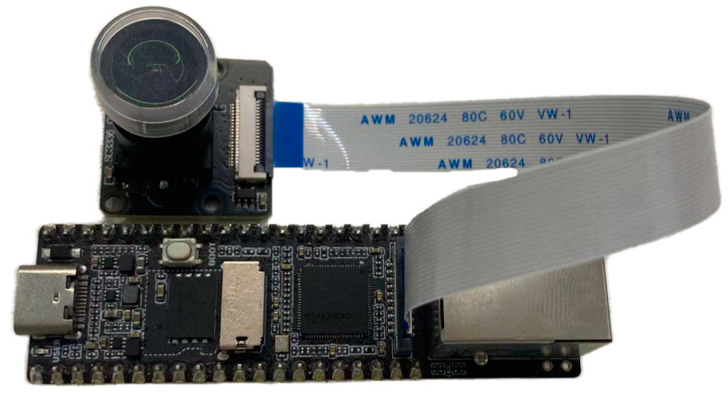
LuckFox Pico Plus.

For edge computing experiments, the LuckFox Pico Plus device, powered by the Rockchip RV1103 chip, was utilized, as shown in Figure 8. The device features a single-core ARM Cortex-A7 32-bit processor with integrated NEON and FPU. Its embedded neural network processing unit (NPU) supports mixed-precision operations (int4, int8, int16) with a peak computational capacity of 0.5 TOPS.

This setup allowed for thorough evaluation of the proposed model, considering both high-performance desktop environments and resource-constrained edge computing scenarios.

The experiment evaluated the model’s performance based on three key metrics: mean average precision (mAP), number of parameters (Params), and frames per second (FPS). These metrics comprehensively assess the model’s performance from the perspectives of detection accuracy, spatial complexity, and processing speed.

Mean average precision (mAP) is a crucial metric for measuring the accuracy of object detection models. It is derived from two foundational metrics: precision and recall.

Precision reflects the proportion of correctly predicted positive samples among all predicted positive samples, indicating the accuracy of the model’s predictions on positive results. Its formula is as follows:

(1) $$P=\frac{TP}{TP+FP}$$

Here, TP (True Positive) represents the number of samples correctly predicted as positive, and FP (False Positive) denotes the number of samples incorrectly predicted as positive.

Recall measures the proportion of correctly predicted positive samples among all actual positive samples, reflecting the model’s ability to detect positive instances. Its formula is as follows:

(2) $$R=\frac{TP}{TP+FN}$$

Here, FN (False Negative) denotes the number of samples incorrectly predicted as negative while being actually positive.

Average precision (AP) is defined as the area under the precision–recall (P-R) curve across different recall values. It is calculated as:

(3) $$AP=\int_{0}^{1}P\left(R\right)\;dR$$

Mean average precision (mAP) is the mean of the AP values across all categories, indicating the model’s detection performance across all classes. A higher mAP value implies better performance. The formula is as follows:

(4) $$mAP=\frac{1}{n}\sum_{i=1}^{n}AP\left(i\right)$$

Here, n represents the total number of categories, and $AP\left(i\right)$ is the average precision for the i-th category.

The number of parameters (Params) refers to the total number of parameters involved during the training process, including weights, biases, and other learnable components. It is an important metric for assessing the spatial complexity of a model. A lower parameter count generally indicates a more lightweight model, which is advantageous for deployment in resource-constrained environments. Frames per second (FPS) measures the model’s inference speed, representing the number of image frames the model can process per second. This metric is a critical indicator of the model’s performance and efficiency, especially in real-time applications. A higher FPS value implies faster processing and better suitability for time-sensitive tasks.

### 3.2. Ablation Study

**Figure 9:**
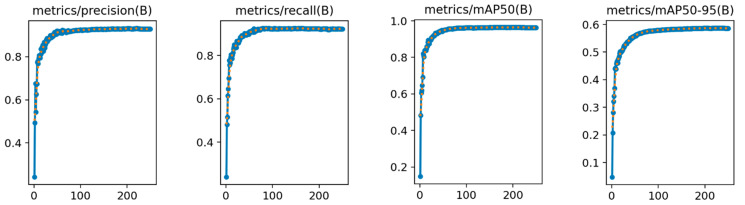
LightYOLO-AppleLeafDx performance indicator curve.

In the analysis of the experimental results, the ablation study plays a crucial role in validating the effectiveness of the proposed improvements. This study primarily evaluates the impact of different improvement components on the performance of the YOLOv8n model in detecting apple leaf diseases. The performance metrics during the model training process are illustrated in Figure 9.

From the results of the ablation experiments presented in Table 3, it is evident that the original YOLOv8n model performs well across various metrics. However, incorporating different improvement modules enhances the model’s performance in key metrics such as precision, recall, and mAP to varying extents. Simultaneously, the parameter count and model size are reduced.

The LightYOLO-AppleLeafDx model, which integrates the Slim-Neck, SPD-Conv, and SAHead modules, achieves the highest scores in precision, recall, and mAP@0.5:0.95. Additionally, it maintains a high inference speed of 107.2 FPS while reducing the parameter count and model size. This result demonstrates that a well-designed combination of modules can significantly improve overall model performance without a substantial increase in computational resource requirements.

**Figure 10:**
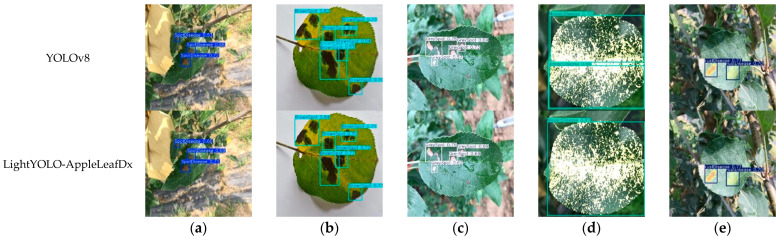
YOLOv8n and LightYOLO-AppleLeafDx detection visualization. (**a**) Spot disease, (**b**) brown spot, (**c**) grey spot, (**d**) mosaic disease, (**e**) rust disease.

The YOLOv8n model and the LightYOLO-AppleLeafDx model were evaluated on the test set, with the detection outcomes presented in Figure 10. The performance metrics for each disease type are as follows: spot disease achieved a precision (P) of 0.905, recall (R) of 0.929, and mAP50 of 0.965; brown spot achieved P = 0.862, R = 0.864, and mAP50 = 0.914; grey spot achieved *p* = 0.944, R = 0.958, and mAP50 = 0.977; mosaic disease achieved *p* = 0.994, R = 1, and mAP50 = 0.995; and rust disease achieved *p* = 0.943, R = 0.863, and mAP50 = 0.973. In summary, the ablation study confirms that the Slim-Neck, SPD-Conv, and SAHead modules positively influence different aspects of the model’s performance. The final LightYOLO-AppleLeafDx model successfully achieves a balance between lightweight design and high accuracy, making it suitable for apple leaf disease detection tasks in resource-constrained environments.

### 3.3. Comparative Experiments with Different Models

In this section, we compare LightYOLO-AppleLeafDx with other mainstream object detection models to evaluate its performance in the apple leaf disease detection task. The models included in the comparison are YOLOv8n, YOLOv5, YOLOv6, and YOLOv7. The experimental results are presented in Table 4.

On the key metric mAP@0.5, LightYOLO-AppleLeafDx achieved a score of 0.965, the highest among all models, outperforming the original YOLOv8n by 0.01. This indicates that LightYOLO-AppleLeafDx offers superior detection accuracy, particularly excelling in apple leaf disease detection tasks. For the more challenging mAP@0.5:0.95 metric, LightYOLO-AppleLeafDx scored 0.587, also outperforming the other models, demonstrating its robust detection capability across objects of varying scales.

In terms of model complexity, as reflected by the number of parameters and floating-point operations (GFLOPs), LightYOLO-AppleLeafDx stands out with parameter counts of only 2.443M and 5.7 GFLOPs, significantly lower than other models. For example, YOLOv6 has 4.500M parameters and 13.1 GFLOPs, indicating a much higher complexity. This demonstrates that LightYOLO-AppleLeafDx effectively reduces model complexity while maintaining high accuracy.

LightYOLO-AppleLeafDx outperforms other YOLO variants across several key metrics, particularly in terms of precision, mAP, and model complexity. These strengths make it the optimal choice for apple leaf disease detection tasks, especially in resource-constrained real-world applications.

### 3.4. Deployment on Edge Devices

**Figure 11:**
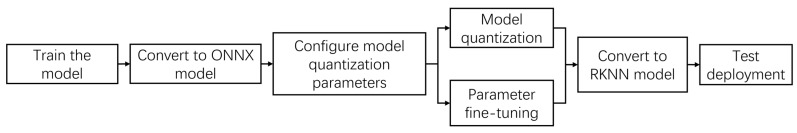
RV1103 deployment flowchart.

To validate the practical effectiveness of the improved model in real-world scenarios, the LightYOLO-AppleLeafDx model was deployed on the LuckFox Pico Plus edge device. The original model, trained and tested on an NVIDIA GPU, cannot be directly utilized on edge devices. To adapt it for the RV1103 chip and enable NPU-accelerated inference, the RKNN-Toolkit2 was employed to convert the PyTorch model into an RKNN model, compatible with the Rockchip NPU. To adapt the PyTorch model for the RV1103 chip and enable NPU-accelerated inference, the RKNN-Toolkit2 was employed. For model quantization, the research followed the official default configuration in the toolkit, including settings such as the quantized data type (asymmetric_quantized-8) and algorithm parameters. The conversion and quantization process is illustrated in Figure 11.

The PyTorch model’s weight file was trained on a server and converted into an ONNX (Open Neural Network Exchange) model, enabling its migration from the training environment to the inference environment.

Model quantization parameters were configured to reduce the model from 32-bit floating-point precision to 8-bit integers. Parameter fine-tuning was performed during this step.

The model was converted into an RKNN format. Feasibility was validated through trial runs on a software simulator using the Python interface of RKNN-Toolkit2. Once verified, the model was deployed on the NPU.

The Rockchip RV1103 utilizes INT8 quantization on its NPU to accelerate inference. This approach reduces computational overhead and lowers power consumption during inference, improving hardware resource efficiency. The deployment achieved the desired functionality at a low cost, balancing computational performance and resource constraints.

Table 5 presents the detection results of the model across different environments. The experiments show that the LightYOLO-AppleLeafDx model achieved the fastest detection speed on the server GPU, reaching 107.2 FPS. Although the trained model architecture and weights remain consistent, edge devices such as the LuckFox Pico Plus leverage NPU-accelerated inference with quantized INT8 precision to meet memory and power limitations, while GPU-based testing utilizes full FP32 precision. When deployed on the RV1103 edge computing device and tested on the RKNN-Toolkit2 simulation platform, the detection speed and accuracy were reduced compared to the server GPU environment. The LightYOLO-AppleLeafDx model demonstrates a 49.2% reduction in inference speed compared to the original YOLOv8n, decreasing from 30.1 NPU-FPS to 14.8 NPU-FPS. Despite this performance decline, the trade-off remains practically acceptable for two key reasons: (1) The achieved inference speed of 14.8 FPS still surpasses the minimum real-time requirement of 10 FPS for agricultural disease monitoring systems, which is suitable given that orchard inspection robots typically operate at speeds below one meter per second; (2) The model’s accuracy improvement, with the mean average precision (mAP) increasing from 95.1% to 95.9%, significantly mitigates the risk of misdiagnosis. This is particularly critical since false negatives in disease detection can result in irreversible crop losses. These considerations align with industry priorities, where accuracy often takes precedence over marginal reductions in latency in precision agriculture applications.

Comparative experiments demonstrated that while LightYOLO-AppleLeafDx achieves a slightly lower NPU-FPS (14.8) than YOLOv5 (17.4) and YOLOv7 (16.3), it maintains superior mAP (95.9%) with reduced computational overhead. As shown in Table 6, the proposed model outperforms all baseline YOLO variants in accuracy while balancing real-time performance on resource-constrained devices.

## 4. Conclusions

This study proposed a lightweight apple leaf disease detection method based on an improved YOLOv8 model. The effectiveness and superior performance of the proposed method were validated through ablation studies and comparative experiments with other models. The main conclusions are as follows:

1. Effectiveness of Model Improvements:

By introducing modules such as Slim-Neck, SPD-Conv, and SAHead, we significantly improved the precision, recall, and mean average precision (mAP) of the YOLOv8n model. The ablation study results demonstrate that both the individual and combined use of these modules enhance detection performance. The final LightYOLO-AppleLeafDx model outperformed the original model across all metrics, achieving a balance between performance and lightweight design.

2. Lightweight and Efficient Design:

The LightYOLO-AppleLeafDx model reduced parameter count and floating-point operations, thereby lowering model complexity while maintaining high detection accuracy. Compared to other mainstream YOLO models, LightYOLO-AppleLeafDx achieved the best results on metrics such as precision and mAP@0.5:0.95, with significantly reduced model size and computational resource requirements. This makes it well suited for deployment on resource-constrained devices.

3. Practical Application Value:

In this study, the model was deployed on the RV1103 edge computing device and tested using network picture transmission to simulate the detection process, thereby validating the model’s accuracy and feasibility. The experimental results showed that this deployment achieved an inference speed of 14.8 FPS and an mAP@0.5 of 95.9% in the simulation tests, demonstrating the model’s strong potential for real-time detection scenarios.

In summary, the proposed LightYOLO-AppleLeafDx model enhances detection performance while achieving lightweight design, demonstrating significant practical application value. Future research could further optimize the model architecture, explore its application on larger-scale datasets, and extend the method to the detection of diseases in other crops.

## Figures and Tables

**Table 1:** Apple leaf disease dataset.

Categories	Label	Dataset	Train Set	Test Set
Spot disease	0	3288	2630	658
Brown spot	1	3096	2477	619
Grey spot	2	2680	2144	536
Mosaic disease	3	2504	2003	501
Rust disease	4	2304	1843	461

**Table 2:** Experimental hyperparameters for deep learning.

Parameter	Value
Epoch	250
Images size	640 × 640
Batch size	32
Learn rate	0.01
Weight_decay	0.0005
Momentum	0.937

**Table 3:** Ablation tests with different modifications.

Method	P	R	mAP@0.5/%	mAP@0.5:0.95/%	Param/M	GFLOPs/G	Model Size/MB	FPS/ ($\mathrm{Frame}\cdot s^{-1}$)
YOLOv8n	0.911	0.921	0.955	0.577	3.157	8.9	6.3	256.7
YOLOv8n + Slim-Neck	0.928	0.926	0.964	0.585	2.799	7.3	5.9	163.8
YOLOv8n + SPD-Conv	0.922	0.932	0.964	0.584	2.789	7.6	5.8	235.8
YOLOv8n + SAHead	0.926	0.924	0.961	0.581	2.868	7	6.0	153.5
YOLOv8n + Slim-Neck + SPD-Conv	0.912	0.940	0.962	0.585	2.582	6.8	5.5	155.6
YOLOv8n + Slim-Neck + SAHead	0.928	0.925	0.963	0.583	2.769	6.6	5.3	121.7
YOLOv8n + SPD-Conv + SAHead	0.924	0.928	0.962	0.582	2.761	6.9	5.3	144.2
LightYOLO-AppleLeafDx	0.930	0.923	0.965	0.587	2.443	5.7	5.2	107.2

**Table 4:** Comparison experiment of different models.

Method	P	R	mAP@0.5/%	mAP@0.5:0.95/%	Param/M	GFLOPs/G	Model Size/MB	FPS/ ($\mathrm{Frame}\cdot s^{-1}$)
YOLOv8n	0.911	0.921	0.955	0.577	3.157	8.9	6.3	256.7
LightYOLO-AppleLeafDx	0.930	0.923	0.965	0.587	2.443	5.7	5.2	107.2
YOLOv5	0.892	0.855	0.945	0.565	2.655	7.8	5.7	145.6
YOLOv6	0.868	0.851	0.935	0.555	4.500	13.1	7.2	110.2
YOLOv7	0.907	0.882	0.950	0.570	3.545	10.4	6.0	135.8

**Table 5:** Detection effect of YOLOv8n and LightYOLO-AppleLeafDx.

Models	Hardware Environment	FPS/ ($\mathrm{Frame}\cdot s^{-1}$)	mAP/%
YOLOv8n	NVIDIA GeForce RTX 2080 Ti	256.7	95.5
	RKNN-Toolkit2-Emulation	53.5	94.8
	RV1103 NPU	30.1	95.1
LightYOLO-AppleLeafDx	NVIDIA GeForce RTX 2080 Ti	107.2	96.5
	RKNN-Toolkit2-Emulation	23.1	95.2
	RV1103 NPU	14.8	95.9

**Table 6:** Detection effect of other models.

Models	NPU-FPS/ ($\mathrm{Frame}\cdot s^{-1}$)	mAP/%
YOLOv8n + Slim-Neck	20.5	0.957
YOLOv8n + SPD-Conv	28.1	0.956
YOLOv8n + SAHead	19.8	0.956
YOLOv8n + Slim-Neck + SPD-Conv	19.8	0.954
YOLOv8n + Slim-Neck + SAHead	15.9	0.953
YOLOv8n + SPD-Conv + SAHead	18.2	0.956
YOLOv5	17.4	0.935
YOLOv6	13.4	0.926
YOLOv7	16.3	0.944

## Data Availability

Data are contained within the article.
